# The effects of liraglutide on expressions of insulin secretion and beta cell survive associated GPCR genes in pancreatic beta cells

**DOI:** 10.55730/1300-0144.5997

**Published:** 2025-03-20

**Authors:** Melikenur TÜRKOL, Türker BİLGEN

**Affiliations:** Department of Nutrition and Dietetics, Faculty of Health Sciences, Tekirdağ Namık Kemal University, Tekirdağ, Turkiye

**Keywords:** Diabetes mellitus, GPCR, insulin secretion, liraglutide

## Abstract

**Background/aim:**

Liraglutide as a long-acting glucagon-like peptide drug has anti-hyperglycemic and antiobesity effects. G protein-coupled receptors (GPCRs) are well-known drug target molecules that conduct critical signaling pathways related with diseases. Research has confirmed the role of many GPCRs genes in the regulation of pancreatic beta cell functions and insulin secretion. Liraglutide dependent expressional changes in GPCR genes may let us determine new drug targets.

**Materials and methods:**

Therefore, we investigated the changes in expression of GPR75, GPR56, GLP1R, M3R, and CB1R genes, which are the GPCR family members, in response to liraglutide treatment in the NIT-1 mouse pancreatic beta cells in this study. Changes at the mRNA levels of these GPCR genes were determined by a qPCR and the ddCT method, and using a control gene and untreated control groups.

**Results:**

We found statistically significant increases at the mRNA levels of GPR75, GPR56, M3R, and CB1R genes with 10nM of liraglutide at min 60, while there was no time and dose-dependent change in all of the genes investigated. We detected that the GLP1R gene expressions were stable amongst different time points and doses of liraglutide, except for a statistically significant decrease in the GLP1R gene expression in response to 1000nM of liraglutide treatment compared to 10nM and 100nM concentrations.

**Conclusion:**

Our results indicate that in vitro liraglutide administration in pancreatic beta cells appears to increase the expressions of GPR75, GPR56, M3R and CB1R genes which have already been related to insulin secretion and beta cell survive. Liraglutide may exert this effect through the GLP1R or other cellular pathways undescribed yet. Combined usage of liraglutide and the specific ligands of GPR75, GPR56, M3R, and CB1R may provide a better response in terms of insulin secretion and beta cell survival, making them good targets for antidiabetic and antiobesity therapy.

## 1. Introduction

Diabetes Mellitus (DM) is a significant public health issue due to its rising prevalence and linked disease’s burden such as obesity, cardiovascular diseases, hypertension, kidney failure, vision loss and foot ulcers. DM is a chronic metabolic disease characterized by the hyperglycemia resulting from defects in insulin secretion, insulin action, or both. Since insulin-secreting pancreatic beta cells loss is a hallmark of both type 1 diabetes (T1D) and type 2 diabetes (T2D) which are the most common forms of DM, in addition to insulin secretion, the studies aiming to compensate for beta cell loss in DM have gained an attraction recently [1].

G protein-coupled receptors (GPCRs), comprising about 800 members of the human genome, are the largest family of cell surface receptors. GPCRs are potential therapeutic targets due to their roles in regulating various physiological processes and their accessibility makes them attractive pharmacological targets [2]. GPR75 has been identified as a 20-Hydroxyeicosatetraenoic acid (20-HETE) receptor [3]. 20-HETE is the omegahydroxylated metabolite of arachidonic acid that affects glucose balance, insulin signaling, and function produced by the Cytochrome P450 (CYP) 4A and 4F enzyme family [5]. It has been shown that the GPR75 has an important role in glycemic control and insulin sensitivity, and loss-of-function mutations of GPR75 are protective against obesity [4]. GPR56, a protein encoded by the ADGRG1 gene, is also known as TM7XN1 and is characterized as a member of the GPCR family [6]. GPR56 is highly expressed in both human and mouse islets, and it has a therapeutic potential for diabetes treatment. Additionally, GPR56 has been reported to play an important role in β-cell functions and survival [7, 8]. Muscarinic Acetylcholine Receptor (M3R) is an acetylcholine receptor and was associated to stimulate insulin secretion [9, 10]. Cannabinoid receptor 1 (CB1R) is a G protein-coupled cannabinoid receptor encoded by the CB1R gene in human [11]. CB1R has been shown to play an active role in the regulation of pancreatic beta cell function and stimulate insulin release [10]. These genes were included in our study due to their defined roles in insulin secretion and β-cell survival.

Furthermore, liraglutide, as a Glucagon-like peptide-1 (GLP-1) analogue and a Glucagon-like peptide-1 receptor (GLP-1R) agonist, has been widely used as an antidiabetic agent worldwide in the treatment of T2D in recent years [12]. As far as it is known, liraglutide exerts its effects through the GLP-1R and effectively mimics the effects of GLP-1 that is an incretin hormone lowering blood glucose levels through the GLP-1R [13]. GLP-1 and its analogues are not only related to the induction of glucose-dependent insulin release [14,15], but also related to the suppression of glucagon release [16], and appetite suppression [17]. At the cellular level, they stimulate pancreatic beta cell replication [18], neogenesis, and differentiation [19]. They inhibit β-cell apoptosis by reducing cellular stress [20, 21]. These lead to a reduction in insulin resistance while improving glucose induced insulin secretion response [22,23]. Therefore, GLP1R response upon liraglutide is much of interest, especially for long-term administrations and unresponsiveness to liraglutide.

This study aimed to elucidate liraglutide’s impact on GPR75, GPR56, GLP1R, M3R, and CB1R gene expressions in the NIT-1 mouse pancreatic beta cells, offering insights into its cellular mechanisms. It also seeks to identify new cellular targets that can be used in combination with liraglutide to improve insulin secretion and β-cell survival as potential antihyperglycemic activity.

## 2. Materials and methods

### 2.1. Cell culture, liraglutide treatment and gene expressions

Liraglutide was obtained from AdipoGen. NIT-1 (ATCC CRL-2055) pancreatic beta cells (passages 29–40) were thawed and cultured in DMEM with 10% FBS, 1% L-glutamine, 100 U/mL penicillin, 100 μg/mL streptomycin, and 100 μg/mL amphotericin B at 37 °C with 5% CO_2_. After seeding 50,000 cells in 6-well plates, they were treated with 10 nM, 100 nM, 1000 nM concentrations of liraglutide by the presence of untreated control groups for each concentration and time points (Liang Wang. Endocrinology 155: 3817–3828, 2014). At the end of 30-, 60-, and 120-min time points, total RNA isolation by the Pure Link kit (Ambion) and cDNA synthesis by the High-Capacity cDNA Reverse Transcription Kit (Thermo Fisher Scientific) were performed. mRNA levels of GPR75, GPR56, GLP1R, M3R, CB1R genes, and a control gene Actin were determined by a real-time qPCR (Applied Biosystems). qRT-PCR primers are listed in Table. The changes in mRNA levels between the groups were assessed using the ddCT method.

*F: Forward; R: Reverse.

### 2.2. Statistical methods

Study data were analyzed at a 95% confidence interval using SPSS 20.0. One-way analysis of variance (ANOVA) identified statistical differences among experimental groups. The posthoc Tukey test was employed for pairwise comparisons if significant differences were detected. Significance was established at p < 0.05 based on the analysis results.

## 3. Results

### Changes in expression of GPR75, GPR56, GLP1R, M3R, CB1R genes

We did not detect any time- and dose-dependent changes in all investigated genes, instead we found a statistically significant increase in mRNA levels of GPR75, GPR56, M3R and CB1R genes at 60 min with 10nM liraglutide and a slight and statistically significant decrease in GLP1R mRNA levels in response to 1000nM liraglutide treatment compared to 10nM and 100nM concentrations at all tested time points, as shown in the figure.

We here investigated the changes in mRNA levels of GPR75, GPR56, GLP1R, M3R, and CB1R genes known as GPCR family members upon in vitro liraglutide treatment in mouse pancreatic beta cells, as alterations in gene expression may let us understand liraglutide’s effects on beta cells, identify mediating molecules, and explore new antidiabetic targets. Despite no time and dose dependent change, we found statistically significant increases in expression levels of GPR75, GPR56, M3R, and CB1R genes with 10 nM of liraglutide at min 60. Moreover, we found that the GLP1R gene expression remained unchanged following various concentrations of liraglutide, except for a slightly but statistically significant decrease in GLP1R mRNA levels in response to 1000 nM liraglutide treatment compared to 10 nM and 100 nM concentrations of liraglutide at all-time points investigated.

## 4. Discussion

GLP-1 increases glucose-induced insulin secretion to lower blood glucose levels. Liraglutide is an acylated human GLP-1 analogue with 97% amino acid similarity to natural glucagon-like peptide-1 (GLP-1). As liraglutide is a GLP-1 analogue, liraglutide reduces blood glucose levels by increasing insulin secretion [24]. GLP-1 is best known for lowering blood glucose levels in diabetics. However, it has also been shown to reduce endoplasmic reticulum stress, regulate autophagy, promote metabolic reprogramming, stimulate anti-inflammatory signaling and alter gene expressions [25]. Ligand binding to the GLP1R initiates the activation of membrane-bound adenylyl cyclase. This initiates a cascade that includes the production of cyclic adenosine monophosphate (cAMP). GLP1R-mediated effects trigger an immediate signaling cascade that can affect insulin release and calcium influx due to rapid posttranslational modifications [26,27]. It may also occur as a result of late-stage or chronic effects that may operate modulation of gene expression or cellular metabolism [28–30]. Although the signaling pathways activated by liraglutide treatment in pancreatic beta cells have been partially elucidated, there is limited information on the gene expression profile that changes in response to liraglutide. While it is expected from the literature that the GLP1R gene expression would increase in liraglutide treated cells, it was observed in our study that the GLP1R gene expressions were stable except 1000 nM liraglutide caused a statistically significant decrease (p < 0.05) compared to both 10 nM and 100 nM of liraglutide. This finding suggests that duration and dose of liraglutide may lead to unexpected changes in gene expression [26,27]. Gençoğlu et al (2019) showed that the ligands CCL5 (GPR75 agonist), ACEA (CBR1 agonist), exenatide (GLP1R agonist) and CCh (M3R agonist) increased insulin secretion in MIN6 mouse insulinoma cells and that the target receptors of these ligands, GPR75, GPR56, M3R, and GLP1R genes were expressed in those cells. The study identified these ligands as potential treatment agents due to their ability to increase insulin secretion [10].

Our results show that GPR75, GPR56, M3R, and CB1R gene expressions are transiently increased by liraglutide treatment. The treatment approaches targeting these genes with their natural ligands may be more effective when used together with liraglutide, especially in patients’ resistant to current antihyperglycaemic therapies. However, this needs to further investigations in both in vitro and in vivo models to be confirmed. Such studies are essential to determine whether combination therapies may offer new and more effective strategies for management of diabetes.

In a study conducted by Amisten et al (2013), the peptide/protein ligands and expression of human pancreatic islet GPCRs were investigated and an atlas of these GPCRs was created. The atlas was developed to investigate the interactions of GPCRs with their endogenous ligands, the mechanisms of regulation of islet hormone secretion, and drug-receptor interactions that may affect insulin release. The study found that the mRNA encoding GPR56 was the most abundant in human pancreatic islets and that it interacts with alpha-1 collagen and activates the RhoA signaling pathway. GPR75 was identified as a new chemokine receptor activated by CCL5 and it was suggested that it may play a role in autoimmune processes by directing lymphocytes to the islets. In addition, GLP1R activation promotes insulin and somatostatin release by increasing cAMP production and enhances glucose-stimulated insulin release by suppressing glucagon release [31].

## 5. Conclusion

In conclusion, our results suggest that these GPR75, GPR56, M3R, and CB1R genes may mediate the previously identified functions of liraglutide in pancreatic beta cells. Given the implications of these findings, GPR75, GPR56, M3R, and CB1R emerge as promising candidates for novel therapeutic targets in the treatment of diabetes and obesity, especially when combined with liraglutide, and in patients less or unresponsive to liraglutide. Targeting GPR75, GPR56, M3R, and CB1R genes may improve liraglutide’s effect. Our findings are important in identifying GPR75, GPR56, M3R, and CB1R genes as targets for more detailed and functional studies. Future research should further explore the mechanistic pathways and clinical implications of these GPCRs.

## Data availability

Not applicable.

## Figures and Tables

**Figure:**
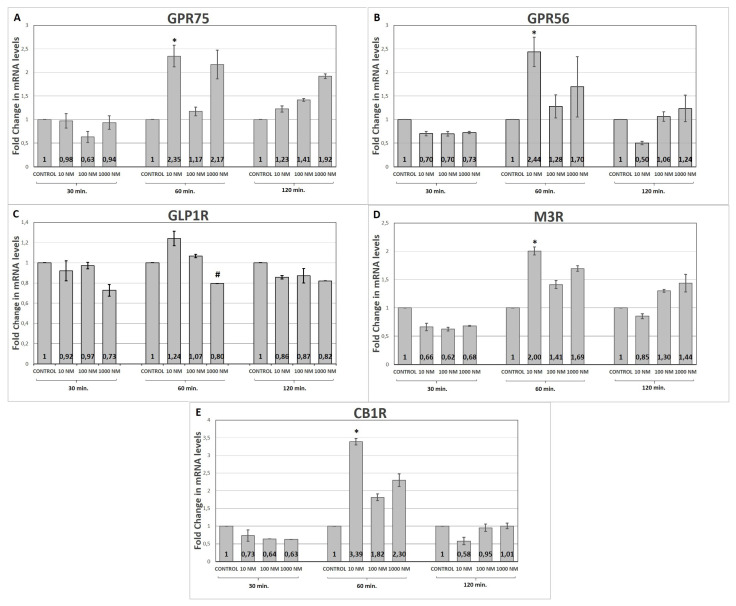
Fold changes in mRNA levels of GPR75 (A), GPR56 (B), GLP1R (C), M3R (D), CB1R (E) genes according to time (30-min, 60-min, 120-min) and liraglutide concentrations (10 nM, 100 nM, and 1000 nM). *Statistically significant increase in mRNA expression of GPR75, GPR56, M3R and CB1R genes with 10nM liraglutide at 60 min compared to 30-and 120-min. Statistical significance is denoted as *<0.05. # Statistically significant decrease in GLP1R mRNA levels with 1000 nM liraglutide treatment compared to 10 nM and 100 nM concentrations at 30, 60 and 120 min (p < 0.05).

**Table:** Primers used for qRT-PCR.

Genes	F/R^*^	Sequences
GPR75	F	5′-CCCTCACCATCATCCTCACT-3′
	R	5′-CCTTCGAGTGACAAACACGA-3′
GPR56	F	5′-GGCTGGAAATCCAGGAGGAC-3′
	R	5′-TCCAAACTGTGCTGCTTTGC-3′
GLP1R	F	5′-GTTTCGGAAATGCTGGGAGC-3′
	R	5′-GTAGGAACTCTGGCAGGTGG-3′
M3R	F	5′-GGGGAACTTAGCCTGTGACC-3′
	R	5′-AGAACAAGATGGCAGGAGCC-3′
CB1R	F	5′-GGAGCAGAGCAGGGGTTC-3′
	R	5′-AACCAACGGGGAGTTGTCTC-3′
ACTIN	F	5′-AGAGAAGATGACGCAGATAATGT-3′
	R	5′-GGTAAAGCTGTAGCCCCGTT-3′

## References

[1] Cummings DM Kirian K Howard G Howard V Yuan Y Consequences of comorbidity of elevated stress and/or depressive symptoms and incident cardiovascular outcomes in diabetes: Results From the REasons for Geographic And Racial Differences in Stroke (REGARDS) Study Diabetes Care 2016 39 101 109 10.2337/DC15-1174 26577418 PMC4876731

[2] Tao YX Molecular chaperones and G protein-coupled receptor maturation and pharmacology Molecular and Cellular Endocrinology 2020 511 10.1016/J.MCE.2020.110862 32389798

[3] Garcia V Gilani A Shkolnik B Pandey V Zhang FF 20-HETE signals through G protein-coupled receptor GPR75 to affect vascular function and trigger hypertension Circulation Research 2017 120 1776 10.1161/CIRCRESAHA.116.310525 28325781 PMC5446268

[4] Hossain S GPR75 deficiency attenuates high fat diet-driven obesity and glucose intolerance NYMC Student Theses Dissertations 2023 55 https://touroscholar.touro.edu/nymc_students_theses/55/

[5] Rocic P Schwartzman ML 20-HETE in the regulation of vascular and cardiac function Pharmacology Therapeutics 2018 192 87 10.1016/J.PHARMTHERA.2018.07.004 PMC6278600 30048707

[6] Langenhan T Aust G Hamann J Sticky signaling—adhesion class G protein–coupled receptors take the stage Science Signaling 2013 6 10.1126/SCISIGNAL.2003825 23695165

[7] Amisten S Atanes P Hawkes R Ruz-Maldonado I Liu B A comparative analysis of human and mouse islet G-protein coupled receptor expression Scientific Reports 2017 1 11 10.1038/srep46600 28422162 PMC5395952

[8] Dunér P Al-Amily IM Soni A Asplund O Safi F Adhesion G Protein-Coupled Receptor G1 (ADGRG1/GPR56) and pancreatic-cell function The Journal of Clinical Endocrinology Metabolism 2016 101 4637 4645 10.1210/jc.2016-1884 27636017

[9] Eglen RM Muscarinic receptor subtypes in neuronal and non-Neuronal cholinergic function Autonomic and Autacoid Pharmacology 2006 26 219 233 10.1111/J.1474-8673.2006.00368.X 16879488

[10] Gençoğlu H Şahin K Jones PM Determining the insulin secretion potential for certain specific G-Protein coupled receptors in MIN6 pancreatic beta cells Turkish Journal of Medical Sciences 2019 49 403 411 10.3906/SAG-1712-147 30761839 PMC7350869

[11] Saito VM Rezende RM Teixeira AL Cannabinoid modulation of neuroinflammatory disorders Current Neuropharmacology 2012 10 166 10.2174/157015912800604515 PMC3386505 23204985

[12] Blonde L Russell-Jones D The safety and efficacy of liraglutide with or without oral antidiabetic drug therapy in type 2 diabetes: An Overview of the LEAD 1–5 Studies Diabetes Obesity and Metabolism 2009 11 3 26 34 10.1111/J.1463-1326.2009.01075.X 19878259

[13] Buse JB Garber A Rosenstock J Schmidt WE Brett JH Liraglutide treatment is associated with a low frequency and magnitude of antibody formation with no apparent impact on glycemic response or increased frequency of adverse events: Results from the liraglutide effect and action in diabetes (LEAD) Trials Journal of Clinical Endocrinology Metabolism 2011 96 1695 1702 10.1210/JC.2010-2822 21450987

[14] Campbell JE Drucker DJ Pharmacology, physiology, and mechanisms of incretin hormone action Cell Metabolism 2013 17 819 837 10.1016/J.CMET.2013.04.008 23684623

[15] Kreymann B Ghatei MA Williams G Bloom SR Glucagon-like Peptide-1 7–36: a physiological incretin in man Lancet 1987 330 1300 1304 10.1016/S0140-6736(87)91194-9 2890903

[16] Nauck MA Wollschläger D Werner J Holst JJ Ørskov C Effects of subcutaneous glucagon-like peptide 1 (GLP-1) in patients with NIDDM Diabetologia 1996 39 1546 1553 10.1007/S001250050613 8960841

[17] Lee YS Shin S Shigihara T Hahm E Liu MJ Glucagon-like peptide-1 gene therapy in obese diabetic mice results in long-term cure of diabetes by improving insulin sensitivity and reducing hepatic gluconeogenesis Diabetes 2007 56 1671 1679 10.2337/DB06-1182 17369525

[18] Friedrichsen B Ni Neubauer N Lee YC Gram VK Blume N Petersen JS Stimulation of pancreatic beta-cell replication by incretins involves transcriptional induction of cyclin D1 via multiple signalling pathways Journal of Endocrinology 2006 188 481 492 10.1677/JOE.1.06160 16522728

[19] Paris M Tourrel-Cuzin C Plachot C Ktorza A Review: Pancreatic beta-cell neogenesis revisited Experimental Diabetes Research 2004 5 111 121 10.1080/15438600490455079 PMC2496878 15203882

[20] Bregenholt S Møldrup A Blume N Karlsen AE Friedrichsen BN The long-acting glucagon-like peptide-1 analogue, liraglutide, inhibits beta-cell apoptosis in vitro Biochemical and Biophysical Research Communications 2005 330 577 584 10.1016/J.BBRC.2005.03.013 15796922

[21] Turrens JF Mitochondrial formation of reactive oxygen species The Journal of Physiology 2003 552 335 344 10.1113/jphysiol.2003.049478 14561818 PMC2343396

[22] Drucker DJ Nauck MA The incretin system: Glucagon-like peptide-1 receptor agonists and dipeptidyl peptidase-4 inhibitors in type 2 diabetes Lancet 2006 368 1696 1705 10.1016/S0140-6736(06)69705-5 17098089

[23] Kalra S Kalra B Kumar S Sharma A Managing insulin resistance: Role of liraglutide Clinical Pharmacology 2010 2 134 10.2147/CPAA.S10496 PMC3262368 22291496

[24] Deacon CF Potential of liraglutide in the treatment of patients with type 2 diabetes Vascular Health Risk Management 2009 5 199 211 10.2147/VHRM.S4039 19436648 PMC2672437

[25] Rowlands J Heng J Newsholme P Carlessi R Pleiotropic effects of GLP-1 and analogs on cell signaling, metabolism, and function Frontiers in Endocrinoogy 2018 9 672 10.3389/FENDO.2018.00672 PMC6266510 30532733

[26] MacDonald PE El-kholy W Riedel MJ Salapatek AMF Light PE The multiple actions of GLP-1 on the process of glucose-stimulated insulin secretion Diabetes 2002 51 434 442 10.2337/DIABETES.51.2007.S434 12475787

[27] Peyot ML Gray JP Lamontagne J Smith PJS Holz GG Glucagon-like Peptide-1 induced signaling and insulin secretion do not drive fuel and energy metabolism in primary rodent pancreatic beta cells PLoS One 2009 4 6221 10.1371/JOURNAL.PONE.0006221 PMC2704866 19593440

[28] Carlessi R Chen Y Rowlands J Cruzat VF Keane KN GLP-1 receptor signalling promotes β-cell glucose metabolism via MTOR-dependent HIF-1α activation Scientific Reports 2017 7 1 13 10.1038/s41598-017-02838-2 28572610 PMC5454020

[29] Cornu M Modi H Kawamori D Kulkarni RN Joffraud M Glucagon-like peptide-1 increases β-Cell glucose competence and proliferation by translational induction of insulin-like growth factor-1 receptor expression Journal of Biological Chemistry 2010 285 10545 10.1074/JBC.M109.091116 PMC2856261 20145256

[30] Rowlands J Cruzat V Carlessi R Newsholme P Insulin and IGF-1 receptor autocrine loops are not required for exendin-4 induced changes to pancreatic β-cell bioenergetic parameters and metabolism in BRIN-BD11 cells Peptides 2018 100 140 149 10.1016/J.PEPTIDES.2017.11.015 29412813

[31] Amisten S Salehi A Rorsman P Jones PM Persaud SJ An atlas and functional analysis of g-protein coupled receptors in human islets of langerhans Pharmacology Therapeutics 2013 139 359 391 10.1016/J.PHARMTHERA.2013.05.004 23694765

